# Mitigating Multipath Bias Using a Dual-Polarization Antenna: Theoretical Performance, Algorithm Design, and Simulation

**DOI:** 10.3390/s17020359

**Published:** 2017-02-13

**Authors:** Lin Xie, Xiaowei Cui, Sihao Zhao, Mingquan Lu

**Affiliations:** Department of Electronic Engineering, Tsinghua University, Beijing 100084, China; xie_lin@foxmail.com (L.X.); zsh_thu@mail.tsinghua.edu.cn (S.Z.); lumq@mail.tsinghua.edu.cn (M.L.)

**Keywords:** dual-polarization, LHCP, short delay multipath, MLE

## Abstract

It is well known that multipath effect remains a dominant error source that affects the positioning accuracy of Global Navigation Satellite System (GNSS) receivers. Significant efforts have been made by researchers and receiver manufacturers to mitigate multipath error in the past decades. Recently, a multipath mitigation technique using dual-polarization antennas has become a research hotspot for it provides another degree of freedom to distinguish the line-of-sight (LOS) signal from the LOS and multipath composite signal without extensively increasing the complexity of the receiver. Numbers of multipath mitigation techniques using dual-polarization antennas have been proposed and all of them report performance improvement over the single-polarization methods. However, due to the unpredictability of multipath, multipath mitigation techniques based on dual-polarization are not always effective while few studies discuss the condition under which the multipath mitigation using a dual-polarization antenna can outperform that using a single-polarization antenna, which is a fundamental question for dual-polarization multipath mitigation (DPMM) and the design of multipath mitigation algorithms. In this paper we analyze the characteristics of the signal received by a dual-polarization antenna and use the maximum likelihood estimation (MLE) to assess the theoretical performance of DPMM in different received signal cases. Based on the assessment we answer this fundamental question and find the dual-polarization antenna’s capability in mitigating short delay multipath—the most challenging one among all types of multipath for the majority of the multipath mitigation techniques. Considering these effective conditions, we propose a dual-polarization sequential iterative maximum likelihood estimation (DP-SIMLE) algorithm for DPMM. The simulation results verify our theory and show superior performance of the proposed DP-SIMLE algorithm over the traditional one using only an RHCP antenna.

## 1. Introduction

It is well known that multipath (MP) effect is one of the main error sources that deteriorate positioning accuracy of Global Navigation Satellite System (GNSS) receivers. With technological advancement in both hardware and software, most of the common-mode errors such as satellite clock error and ionosphere error have been eliminated by methods like dual-frequency and differential techniques [1]. However, the multipath, which depends on the surrounding environment where the receiver is situated, is still one of the dominant error sources in applications that require high accuracy [2].

In the past decades, researchers and manufacturers have proposed a number of multipath mitigation techniques and they can be grouped into three categories according to the applied stages along the processing of the signal:

(1) Pre-receiver techniques suppress the reception of the MP signals via spatial diversity [3,4,5]. The spatial diversity is easy to understand for the fact that the line-of-sight (LOS) and MP signals propagate in different paths and arrive at the receiver antenna from different directions. Hence, the receiver antennas are designed to have either fixed or configurable radiation patterns to enhance the reception of the LOS signal while suppressing the reception of the MP signals. However, these techniques have either bulky antennas or complex hardware.
(2) Receiver baseband signal processing techniques reduce the MP bias by discriminator design [6] or by estimating the parameters of the LOS signal from the compound of LOS and MP signals based on the estimation theory. The techniques based on discriminator design are adequate in mitigating medium to long delay multipaths, but the short delay multipath, which is one of the most challenging multipaths to mitigate, makes most of the algorithms in this category less effective [7]. The path parameter estimation techniques are often based on maximum likelihood estimation (MLE) such as the multipath mitigation technology (MMT) [8,9,10], the multipath estimating delay lock loop (MEDLL) [11,12], the Newton method-based fast iterative maximum-likelihood algorithm (FIMLA) [13], and the space-alternating generalized expectation maximization (SAGE) algorithm [14,15]. However, these methods are sensitive to the signal models and require high amount of computation.
(3) Post-receiver or measurement domain techniques exploit the characteristics of the MP interfered measurements to average or compensate the multipath bias [16,17]. They need prior knowledge about the multipath and are restricted to specific multipath environments [18].

Recently, dual-polarization antenna technology has become a research hotspot, for it provides an additional diversity to distinguish the LOS signal from the MP signals. The polarization diversity is exploited based on the fact that the GNSS satellite transmits right hand circular polarized (RHCP) signal and it turns partly to a left hand circular polarized (LHCP) signal upon reflection. The traditional receiver antenna is designed to be sensitive to RHCP signal; hence, it receives the LOS signal and the RHCP component of the MP signal, while the dual-polarization antenna has an additional LHCP channel to receive the LHCP component of the MP signal. Dual-polarization antenna technology has been used in various GNSS remote sensing applications, e.g., reflected signal analysis and altimetry [19,20], precipitation detection [21], and vegetation sensing [22]. With the development of antenna hardware technology, the dual-polarization antenna has similar size with the RHCP antenna, which makes it easy to extend its applications, without extensively increasing the complexity, in the cases where there used to be an RHCP antenna, e.g., the survey receiver, vehicle navigation, and reference station. In the past decade, numbers of multipath mitigation techniques using dual-polarization antennas have been proposed and they can also be grouped into the aforementioned three categories. 

In addition to the spatial diversity, dual-polarization antennas provide polarization diversity to distinguish LOS and MP signals to enhance the suppression of the received MP signals. Aloi uses a pair of orthogonal dipoles instead of the directive antenna to suppress the ground-reflected multipath [23]. Brenneman et al. use an array of LHCP antennas for better angle of arrival (AOA) estimations of multipath, which are used to suppress the received MP signals in the array of RHCP antennas [24]. Both of the works report benefits of using dual-polarization antennas to improve the multipath mitigation performance in the spatial domain. However, whether the usage of dual-polarization antennas always contributes to multipath mitigation in the spatial domain remains a question.

In baseband signal processing, the dual-polarization antenna produces additional information about the reflected MP signal in the LHCP channel to enhance multipath parameter estimation. Wendler et al. utilize dual-polarization antennas in a statistical signal model to make the LOS signal easier to be distinguished from the MP signals in both time and spatial domains especially in cases where signals are highly spatially correlated [25]. However, in the time domain, he did not discuss if the utilization of dual-polarization antennas improves the multipath mitigation regardless of the relative time delay of multipath. Authors of [26,27,28,29] also integrate the dual-polarization technique into the state-of-the-art baseband signal processing algorithms and obtain better multipath mitigation performance, but the question is still unsettled.

The post-receiver techniques based on dual-polarization antennas have more measurements extracted from the additional LHCP channel to detect multipath or reduce the multipath bias, e.g., the estimated carrier power to noise ratios (CNRs) of the RHCP and LHCP channels in a dual-polarization antenna are combined to detect multipath [30,31,32,33]. It is worth noting that, in [30], Groves et al. conclude the cases where the dual-polarization antenna can or cannot help to detect multipath based on the CNR difference between RHCP and LHCP channels. However, this conclusion does not take the relative delay between the LOS and MP signals into consideration.

To summarize, multipath mitigation techniques report performance improvement when using dual-polarization antennas. Meanwhile, as mentioned in [23,28,30], their DPMM algorithms are not always effective. However, few of these studies focus on the cases in which the multipath mitigation based on parameter estimation can or cannot benefit from dual-polarization antenna technology. On the other hand, the dual-polarization antenna technology requires high-quality circular polarization antennas, additional LHCP channel output, doubled correlators, and increased computational load. These are the weaknesses of DPMM technology. Therefore, before the adoption of dual-polarization technology or the design of DPMM algorithms, the fundamental question about the conditions under which the DPMM can outperform that of single-polarization and its theoretical performance improvement should be answered.

In this paper, we model the received signal by a dual-polarization antenna concerning the polarization states of both the antennas and the reflected signals. Based on the typical parameters of the GNSS dual-polarization antenna and the electrical properties of the reflective materials, we analyze the characteristics of the received signals and classify them into strong LHCP signal and weak LHCP signal cases. After that we propose a MLE algorithm for multipath parameter estimation of a dual-polarization antenna to evaluate the performance of DPMM. By comparing the performance of the DPMM algorithm with that of the single-polarization-intended MLE algorithms in different received signals cases based on Monte Carlo simulations, we answer the fundamental question in some typical cases. Inspired by the effective conditions, we propose a computationally reasonable DPMM algorithm which shows superior performance in mitigating short delay multipath over the RHCP-based algorithm.

The rest of this paper is organized as follows: Section 2 introduces the received signal model of the dual-polarization antenna concerning both the reflection and the polarization mismatch loss on reception. Based on the analysis of the power of the received signal components in the LHCP antenna, we generate four typical cases for multipath mitigation evaluation; in Section 3, we compare the proposed maximum likelihood estimator (ML estimator), which is suitable for the dual-polarization antenna, with other signal-polarization-intended ones and find the effective condition and its capability of mitigating short delay multipath; a DPMM algorithm concerning the effective conditions with reasonable complexity is proposed in Section 4; the simulations in Section 5 test the performance of the proposed algorithm in both normal and critical scenarios; conclusions are given in Section 6.

## 2. Received Signal Model of a Dual-Polarization Antenna in the One LOS and One Reflected Path Environment

**Figure 1:**
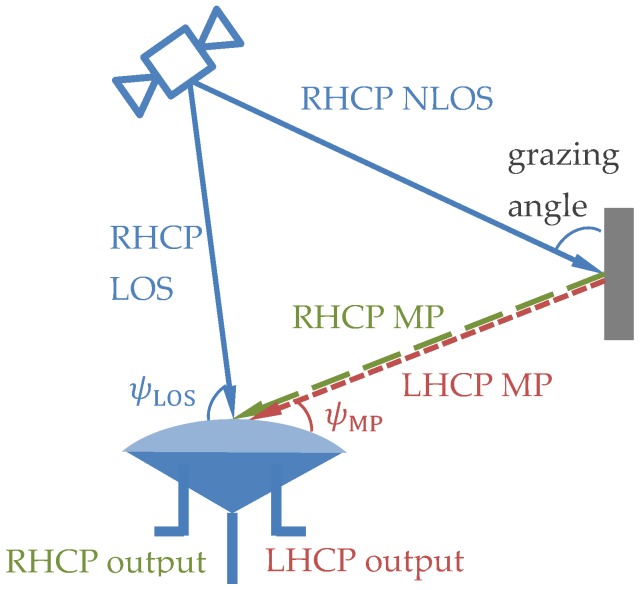
The components of the received signal by a dual-polarization antenna.

A dual-polarization antenna is a single antenna whose internal elements are combined in two different ways to produce RHCP-sensitive and LHCP-sensitive outputs [32]. As Figure 1 shows, when the NLOS signal is reflected, it turns to an elliptical polarized (EP) wave; the dual-polarization antenna receives the RHCP LOS signal and both the RHCP and LHCP components of the reflected EP signal.

### 2.1. Received Signal Model

Ideally, the RHCP antenna receives the RHCP component of the mixture of the LOS and MP signals, while the LHCP antenna receives the LHCP component of the MP signals. However, a practical RHCP or LHCP antenna has finite amplitude attenuation to the signals with an opposite polarization state, thus, it receives both RHCP and LHCP signals [28]. The attenuation or the polarization mismatch loss has relevance to the axial ratios (ARs) of the antennas and the signals, which defines the polarization state of an electric field as Equation (1) gives [34]:

(1) $$R=\frac{E_{R}+E_{L}}{E_{R}-E_{L}}\;\mathrm{or}\;R\left(\mathrm{dB}\right)=20\;\times \;\log10\left(\left|R\right|\right)$$

where $E_{R}$ and $E_{L}$ are the amplitudes of the RHCP and LHCP components, respectively. If R>0, it is a right hand elliptical polarized (RHEP) wave and R<0 indicates a left hand elliptical polarized (LHEP) wave. The AR of the polarization ellipse of the antenna is also defined in the same way.

When the ARs of the signal and antenna are different, polarization mismatch loss occurs as given in Equation (2), which is used in [18,34]:

(2) $$p(R_{A},R_{S},\Delta \tau )=\frac{1}{2}+\frac{4R_{S}R_{A}+(R_{S}^{2}-1)(R_{A}^{2}-1)\times \cos2\Delta \tau }{2(R_{S}^{2}+1)(R_{A}^{2}+1)}$$

where $R_{S}$ and $R_{A}$ are the ARs of the signal and the antenna, respectively. Δτ is the relative tilt angle between the major axes of the polarization ellipses of the signal and the receiver antenna. The AR of the antenna $R_{A}\left(\psi ,\varphi \right)$ is a function of the elevation angle ψ and the azimuth angle φ. The AR of the LOS signal depends on the AR of the satellite antenna which is no worse than 1.8 dB [35] while the AR of the MP signal is mainly determined by reflection. Equation (3) shows the complex circular reflection coefficients represented by the horizontal and vertical reflection coefficients:

(3) $$\begin{array}{l} \Gamma_{o}=\left|\Gamma_{o}\right|e^{-j∠\Gamma_{o}}=\frac{\Gamma_{h}+\Gamma_{v}}{2}\\ \Gamma_{x}=\left|\Gamma_{x}\right|e^{-j∠\Gamma_{x}}=\frac{\Gamma_{h}-\Gamma_{v}}{2} \end{array}$$

where $\left|\Gamma_{o}\right|$ and $∠\Gamma_{o}$ are the relative amplitude and the phase change of the co-polar reflection coefficient, respectively, while $\left|\Gamma_{x}\right|$ and $∠\Gamma_{x}$ are the corresponding cross-polar terms. The derivation of the horizontal $\Gamma_{h}$ and vertical $\Gamma_{v}$ reflection coefficients can be found in [1,18].

For simplicity, we assume there is a single one-bounce reflected multipath hereinafter. The model of the complex baseband signal that a dual-polarization antenna receives is given in Equation (4):

(4) $$\begin{array}{l} x_{\mathrm{RHCP}}(t)=a_{0}e^{j\beta_{0}}p(R_{R}(\psi_{0},\varphi_{0}),R_{\mathrm{LOS}},\Delta \tau_{0})s(t-t_{0})\\ \;+a_{1}e^{j\beta_{1}}\left|\Gamma_{o}\right|e^{-j∠\Gamma_{o}}p(R_{R}(\psi_{1},\varphi_{1}),R_{\mathrm{MP}},\Delta \tau_{1})s(t-t_{1})+w_{R}(t)\\ x_{\mathrm{LHCP}}(t)=a_{0}e^{j\beta_{0}}p(R_{L}(\psi_{0},\varphi_{0}),R_{\mathrm{LOS}},\Delta \tau_{0})s(t-t_{0})\\ \;+a_{1}e^{j\beta_{1}}\left|\Gamma_{x}\right|e^{-j∠\Gamma_{x}}p(R_{L}(\psi_{1},\varphi_{1}),R_{\mathrm{MP}},\Delta \tau_{1})s(t-t_{1})+w_{L}(t) \end{array}$$

where $s\left(t-t_{0}\right)$ and $s\left(t-t_{1}\right)$ are the baseband signals of LOS and MP, respectively, a and β are the amplitude and the carrier phase, respectively. The subscript 0 represents the LOS signal and 1 represents the MP signal. $p\left(R_{A},R_{S},\Delta \tau \right)$ is the polarization mismatch loss in Equation (2), $R_{R}\left(\psi ,\varphi \right)$ is the AR of the RHCP antenna at a given direction (ψ,φ) and $R_{L}\left(\psi ,\varphi \right)$ is that of the LHCP antenna; $R_{\mathrm{LOS}}$ is the AR of the LOS signal and $R_{\mathrm{MP}}$ is that of the MP signal which is a function of the grazing angle and the electrical property of the reflective surface. $w_{R}\left(t\right)$ and $w_{L}\left(t\right)$ are the independent additive white Gaussian noise.

Equation (4) can be rewritten in a compact form in Equation (5):

(5) $$\begin{array}{l} x_{\mathrm{RHCP}}(t)=A_{0}e^{j\theta_{0}}s(t-t_{0})+A_{1}e^{j\theta_{1}}s(t-t_{1})+w_{R}(t)\\ x_{\mathrm{LHCP}}(t)=B_{0}e^{j\theta_{0}^{\prime }}s(t-t_{0})\;+B_{1}e^{j\theta_{1}^{\prime }}s(t-t_{1})+w_{L}(t) \end{array}$$

where the amplitudes and phases considering reflection and reception are rewritten in Equation (6).

(6) $$\begin{array}{ll} A_{0}=a_{0}p(R_{R}(\psi_{0},\varphi_{0}),R_{\mathrm{LOS}},\Delta \tau_{0}) & \theta_{0}=\beta_{0}\\ A_{1}=a_{1}\left|\Gamma_{o}\right|p(R_{R}(\psi_{1},\varphi_{1}),R_{\mathrm{MP}},\Delta \tau_{1}) & \theta_{1}=\beta_{1}-∠\Gamma_{o}\\ B_{0}=a_{0}p(R_{L}(\psi_{0},\varphi_{0}),R_{\mathrm{LOS}},\Delta \tau_{0}) & \theta_{0}^{\prime }=\beta_{0}\\ \;B_{1}=a_{1}\left|\Gamma_{x}\right|p(R_{L}(\psi_{1},\varphi_{1}),R_{\mathrm{MP}},\Delta \tau_{1}) & \theta_{1}^{\prime }=\beta_{1}-∠\Gamma_{x} \end{array}.$$

Now we have modeled the signal received by a dual-polarization antenna considering both reflection and reception. In the next subsection, we will calculate the relative power of each component with respect to $A_{0}^{2}$, to analyze the characteristics of the received signal.

### 2.2. Classification of the Received Signals

The signal model in Equation (4) or (5) has a similar form with those in [26,28]. However, models in the references do not consider the restriction of the signal amplitudes in LHCP antenna. If we use the typical AR values of signals and antennas, i.e., $R_{\mathrm{LOS}}=1.8\;\mathrm{dB}$ [35] and $R_{L}\left(\psi_{0},\varphi_{0}\right)=2\;\mathrm{dB}$ [34] and then substitute Equations (1) and (2), into $B_{0}$ in Equation (6), the power of the LOS signal ($B_{0}^{2}$) received by the LHCP antenna ranges from −39.0 dB ($\Delta \tau_{0}=90^{{}^{\circ}}$) to −13.3 dB ($\Delta \tau_{0}=0^{{}^{\circ}}$) as $A_{0}^{2}$ is normalized to 0 dB. Palamartchouk also reports that their receiving antenna provides cross-polarization selectivity at the level of approximately 15 dB or better for the reception of a signal coming from the zenith [33], which corresponds with our calculation. 

**Figure 2:**
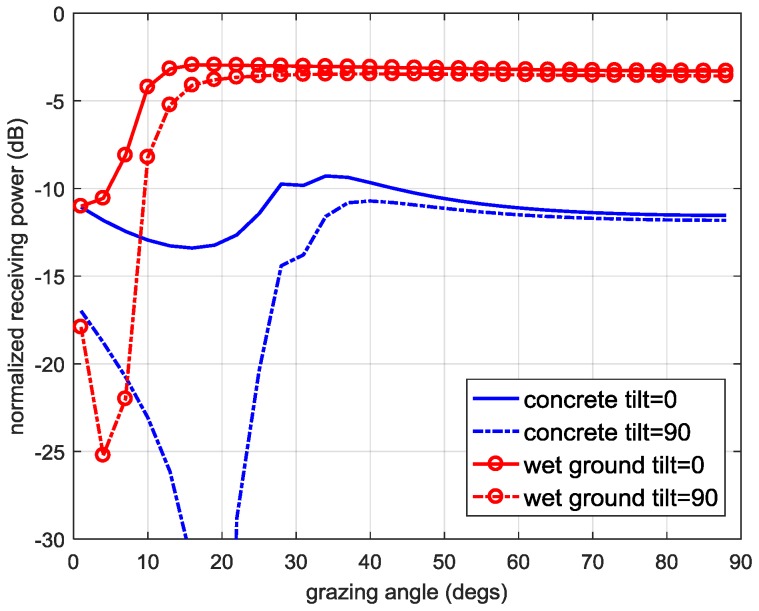
The relative power of the one-bounce multipath received by the LHCP antenna when different types of materials are used. The AR of the MP signal before reflection is 1.8 dB, and $R_{L}\left(\psi_{1},\varphi_{1}\right)=4\;\mathrm{dB}$. The received MP signal power is normalized to that of the LOS signal.

Before determination of $B_{1}^{2}$, we assume that the power of the MP signal is no higher than that of the LOS signal, and the AR of the MP signal before reflection is the same with the LOS signal, i.e., 1.8 dB, and $R_{L}\left(\psi_{1},\varphi_{1}\right)=4\;\mathrm{dB}$ (different AR values from 2 dB to 10 dB alter little to the range of the received MP signal power in LHCP antenna). By substituting Equations (1)–(3), the typical parameters of antennas as assumed above, and the electrical properties of the reflective materials given in [1] into $B_{1}$ in Equation (6), Figure 2 illustrates the relative power of the MP signal ($B_{1}^{2}$) received by the LHCP antenna. When the reflected material is wet ground, the received MP signal in the LHCP antenna will not exceed −3 dB as the red circle lines in Figure 2 illustrates; if it is reflected by concrete, this upper bound is approximately −9 dB as the blue lines show. Based on the results of the reflection of these two typical materials, generally we assume that the power of $B_{1}^{2}$ ranges from −40 dB to −3 dB as $A_{0}^{2}$ is normalized to 0 dB (it is worth noting that if there is no multipath, the lower bound of $B_{1}^{2}$ can be minus infinity and if it is reflected by a metal surface, the received power can reach as high as about 0 dB in theory). Similar with the determination of $B_{1}^{2}$, the range of $A_{1}^{2}$ is from −40 dB to 0 dB.

To sum up, the relative power of LOS and MP signals received by a dual-polarization antenna can be concluded in Table 1 below.

In this paper we investigate how the additional LHCP antenna in a dual-polarization antenna contributes to mitigating the multipath that interferes with the LOS signal in RHCP antenna, thus, the relative power of the MP signal ($A_{1}^{2}$) in the RHCP channel is set constantly to −6 dB for evaluation. Concerning the variation of both $B_{0}^{2}$ and $B_{1}^{2}$, we choose the maximum receivable power of the LOS signal in LHCP channel, i.e., −15 dB, as the primary factor to discuss the possible received signal cases:

- Weak LHCP signal case: $B_{1}^{2}$ is lower than −15 dB.
- Strong LHCP signal case: $B_{1}^{2}$ is higher than −15 dB.

We choose some typical values to cover the range of the power of $B_{0}^{2}$ and $B_{1}^{2}$. Table 2 shows the specific power level of $B_{0}^{2}$ and $B_{1}^{2}$ with which we continue our analysis in the next section. For convenience, we define the dual-polarization gain (*DPG*) of a multipath as $DPG=\frac{A_{1}^{2}+B_{1}^{2}}{A_{1}^{2}}$, which is the ratio of the total power of the MP signal received by a dual-polarization antenna over that by an RHCP antenna, particularly to describe the contribution of the LHCP antenna in increasing the received power of the multipath signal over an RHCP antenna. *DPG* = 2 is equivalent to $B_{1}^{2}=A_{1}^{2}$, which means the LHCP antenna receives as much of the MP signal power as that of the RHCP antenna.

## 3. Theoretical Performance of DPMM Using MLE under One LOS and One Reflected Path Environment

In this section, we extend the ML estimator from single-polarization to dual-polarization and combine it with the Monte Carlo (MC) simulation to evaluate the theoretical performance of DPMM under the one LOS and one reflected path environment. We compare it with those ML estimators designed for the RHCP antenna, e.g., the one-path ML estimator and the two-path MMT algorithm [9] under the multipath environments defined by the received signal cases in Table 2, to find out: (1) in which condition the ML estimator based on a dual-polarization antenna can outperform that based on an RHCP antenna and (2) how much the performance improvement is under this condition.

### 3.1. Maximum Likelihood Estimators

Here we propose the ML estimator for the time of arrival (TOA) estimation of the LOS signal using a dual-polarization antenna whose signal model is given by Equation (5). 

The data in RHCP and LHCP channels are synchronously sampled on $\left[0,T_{s}\right]$ at the Nyquist rate $T^{-1}$ which yields the discrete time signal model in Equation (7):

(7) $$\begin{array}{l} x_{\mathrm{RHCP}}(nT)=A_{0}e^{j\theta_{0}}s(nT-t_{0})+A_{1}e^{j\theta_{1}}s(nT-t_{1})+w_{R}(nT)\\ x_{\mathrm{LHCP}}(nT)=B_{0}e^{j\theta_{0}^{\prime }}s(nT-t_{0})\;+B_{1}e^{j\theta_{1}^{\prime }}s(nT-t_{1})+w_{L}(nT) \end{array}$$

where n=1,2,3,…,N is the sample point number. The noise components of distinct samples are independent; hence, the log-likelihood function of MLE to be maximized is easy to obtain in Equation (8):

(8) $$\ln p(\eta )=\ln\frac{1}{{\left(2\pi \delta^{2}\right)}^{N}}-\frac{1}{2\delta^{2}}\left(\begin{array}{l} \;\sum_{n=1}^{N}\left\{\begin{array}{l} {\left[x_{\mathrm{RHCP}}\left(nT\right)-A_{0}e^{j\theta_{0}}s(nT-t_{0})-A_{1}e^{j\theta_{1}}s(nT-t_{1})\right]}^{H}\\ {}*\left[x_{\mathrm{RHCP}}\left(nT\right)-A_{0}e^{j\theta_{0}}s(nT-t_{0})-A_{1}e^{j\theta_{1}}s(nT-t_{1})\right] \end{array}\right\}\\ +\sum_{n=1}^{N}\left\{\begin{array}{l} {\left[x_{\mathrm{LHCP}}\left(nT\right)-B_{0}e^{j\theta_{0}^{\prime }}s(nT-t_{0})-B_{1}e^{j\theta_{1}^{\prime }}s(nT-t_{1})\right]}^{H}\\ {}*\left[x_{\mathrm{LHCP}}\left(nT\right)-B_{0}e^{j\theta_{0}^{\prime }}s(nT-t_{0})-B_{1}e^{j\theta_{1}^{\prime }}s(nT-t_{1})\right] \end{array}\right\} \end{array}\right)$$

where ${\left[\;\right]}^{H}$ denotes the Hermitian transpose and $\delta^{2}$ is the variance of the white noise. By substituting the product of the amplitude and exponential term as is done in MMT [9] by Equation (9):

(9) $$x_{\mathrm{RHCP}}\left(nT\right)=x_{I}\left(nT\right)+jx_{Q}\left(nT\right)\;x_{\mathrm{LHCP}}\left(nT\right)=y_{I}\left(nT\right)+jy_{Q}\left(nT\right)\;A_{0}e^{j\theta_{0}}=a+jb\;A_{1}e^{j\theta_{1}}=c+jd\;B_{0}e^{j\theta_{0}^{\prime }}=e+jf\;B_{1}e^{j\theta_{1}^{\prime }}=p+jq$$

the maximization of Equation (8) is equivalent to the minimization of the objective function in Equations (10)–(12) with respect to the parameters $\eta =\left[t_{0},t_{1},\;a,b,c,d,e,f,p,q\right]$ to be estimated:

(10) $$Ο\left(\eta \right)=\ln C+\frac{1}{2\sigma^{2}}\left[{Ο}_{\mathrm{RHCP}}\left(\eta \right)+{Ο}_{\mathrm{LHCP}}\left(\eta \right)\right]$$

(11) $$\begin{array}{l} O_{\mathrm{RHCP}}(\eta )=(a^{2}+b^{2}+c^{2}+d^{2})R_{\mathrm{ss}}(0)+2(ac+bd)R_{\mathrm{ss}}(t_{1}-t_{0})\\ \;-2aR_{\mathrm{sxI}}(t_{0})-2bR_{\mathrm{sxQ}}(t_{0})-2cR_{\mathrm{sxI}}(t_{1})-2dR_{\mathrm{sxQ}}(t_{1}) \end{array}$$

(12) $$\begin{array}{l} O_{\mathrm{LHCP}}(\eta )=(e^{2}+f^{2}+p^{2}+q^{2})R_{\mathrm{ss}}(0)+2(ep+fq)R_{\mathrm{ss}}(t_{1}-t_{0})\\ \;-2eR_{\mathrm{syI}}(t_{0})-2fR_{\mathrm{syQ}}(t_{0})-2pR_{\mathrm{syI}}(t_{1})-2qR_{\mathrm{syQ}}(t_{1}) \end{array}$$

where $R_{\mathrm{ss}}(t_{1}-t_{0})=\sum_{n=1}^{N}s(nT-t_{0})\times s(nT-t_{1})$ is the auto-correlation function (ACF) of the local code wave. $R_{\mathrm{sxI}}(t)=\sum_{n=1}^{N}s(nT-t)\times x_{I}(nT)$ and $R_{\mathrm{sxQ}}(t)=\sum_{n=1}^{N}s(nT-t)\times x_{Q}(nT)$ are the in-phase and quadrature (I/Q) cross-correlation functions (CCFs) of the RHCP channel, respectively while $R_{\mathrm{syI}}(t)$ and $R_{\mathrm{syQ}}(t)$ are the corresponding LHCP terms. $\ln C=\frac{1}{2\sigma^{2}}\sum_{n=1}^{N}\left[{\left|x_{\mathrm{RHCP}}\left(nT\right)\right|}^{2}+{\left|x_{\mathrm{LHCP}}\left(nT\right)\right|}^{2}\right]+N\ln\left(2\pi \sigma^{2}\right)$ is a constant term irrelevant to the minimization. Both the ACF and CCFs have high resolution within the range from −1 to +1 code chip. The $R_{\mathrm{ss}}\left(t\right)$ can be calculated offline and $R_{\mathrm{sxI}}\left(t\right)$,$\;R_{\mathrm{sxQ}}\left(t\right)$, $R_{\mathrm{syI}}\left(t\right)$, $R_{\mathrm{syQ}}\left(t\right)$ are generated by N points of data using signal compression (SC) technology [36]. Therefore, for each pair of $\left[t_{0},\;t_{1}\right]$, the values of ACF and CCFs are known, and the minimization of Ο(η) with respect to the rest parameters [a,b,c,d,e,f,p,q] becomes a quadratic optimization problem that can be easily solved. By linearly searching the space of $\left[t_{0},t_{1}\right]$, we obtain the minimum value of Ο(η) and the optimal TOA estimation $\hat{t_{0}}$ of the LOS signal. The optimization method are similar with that in MMT and we add some constraints [37] to accelerate and improve the algorithm. For convenience, we name the signal model denoted by Equation (7) as “R2L2” and the ML estimator we have derived in Equations (10)–(12) as “DP-R2L2-MLE” hereinafter.

For the purpose of finding the multipath mitigation improvement of the dual-polarization antenna technology over the single-polarization one, we compare the DP-R2L2-MLE with other ML estimators that use other signal models:

- RHCP-R1-MLE: the widely used one-path ML estimator that assumes only the LOS signal in the RHCP antenna. It models the LOS signal in the RHCP channel in Equation (7) and the log-likelihood function is a special case in DP-R2L2-MLE, thus, the terms ${Ο}_{\mathrm{LHCP}}\left(\eta \right)$ in Equation (10) and [c,d] in Equation (11) are zeros. We name this signal model as “R1” and the ML estimator as “RHCP-R1-MLE”.
- RHCP-R2-MLE: the two-path ML estimator or the two-path MMT estimator that models the LOS and MP signals in the RHCP antenna. Its model has only the $x_{\mathrm{RHCP}}\left(nT\right)$ term in Equation (7), hence, the term ${Ο}_{\mathrm{LHCP}}\left(\eta \right)$ in Equation (10) is zero. We name this signal model as “R2” and the corresponding ML estimator as “RHCP-R2-MLE”.
- DP-R2L1-MLE: in Strong LHCP Signal Case 2, $B_{0}$ is much smaller than $B_{1}$, hence, $B_{0}$ can be ignored to yield a modified signal model as Equation (13) shows. In this model, the terms [e,f] in Equation (12) are zeros and we denote the model in Equation (13) as “R2L1” and the corresponding ML estimator as “DP-R2L1-MLE”.
  (13) $$\begin{array}{l} x_{\mathrm{RHCP}}(nT)=A_{0}e^{j\theta_{0}}s(nT-t_{0})+A_{1}e^{j\theta_{1}}s(nT-t_{1})+w_{R}(nT)\\ x_{\mathrm{LHCP}}(nT)=\;B_{1}e^{j\theta_{1}^{\prime }}s(nT-t_{1})+w_{L}(nT) \end{array}$$

### 3.2. The Root Mean Square Error of the ML Estimators

In this subsection we evaluate the ML estimators with MC simulations in each received signal case as listed in Table 2. The baseband I/Q signal is the GPS L1 coarse/acquisition (C/A) signal, which is generated according to Equation (5) with a CNR of 42 dB-Hz. The double-side bandwidth of the pre-correlation filter is 16 MHz. The I/Q data are sampled in 32 MHz for 100 ms each time to generate the ACF and CCFs for ML estimators. Here we define the short delay range as when the delay of multipath, relative to the LOS signal, is from 0 to the inverse of the bandwidth of the signal, i.e., from 0 to 62.5 ns or 0 to 18.75 m.

**Figure 3:**
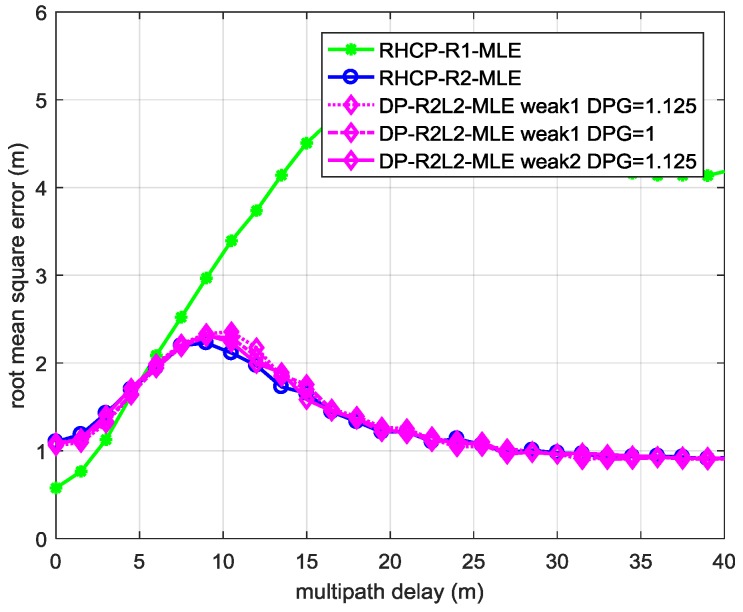
The RMSE of the TOA estimation of the LOS signal using MLE with different models in the two-path environments defined by Weak LHCP Signal Cases 1 and 2 in Table 2.

Figure 3 shows the root mean square error (RMSE) of the TOA of the LOS signal $\hat{t_{0}}$ of each ML estimator as a function of the relative delay of the MP signal to the LOS signal in the two-path environments defined by Weak LHCP Signal Cases 1 and 2 in Table 2. The performance of DP-R2L2-MLE (in magenta diamonds lines) under these environments is almost the same as that of RHCP-R2-MLE (in blue circles line), which is based on a single-polarization antenna. 

**Figure 4:**
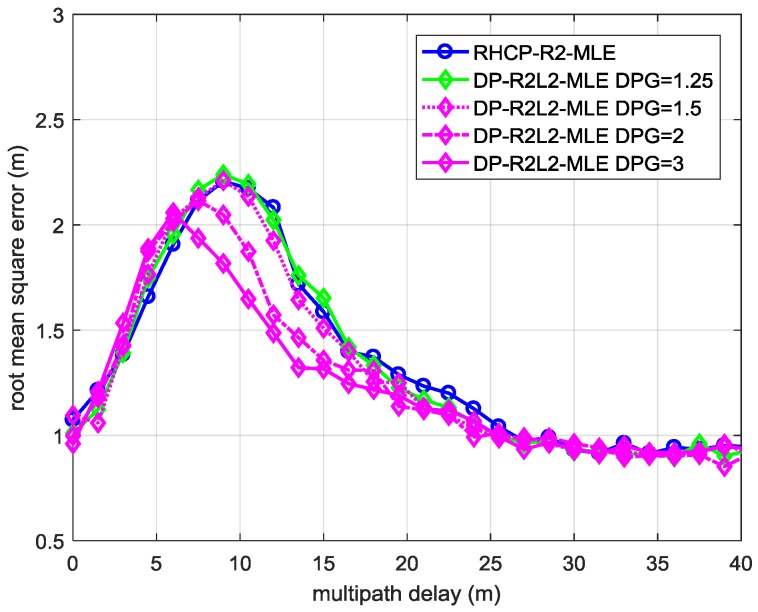
The RMSE of the TOA estimation of LOS signal using MLE with different signal models in the two-path environments defined by Strong LHCP Signal Case 1 in Table 2.

Figure 4 illustrates the RMSEs of $\hat{t_{0}}$ estimated by ML estimators in Strong LHCP Signal Case 1 defined in Table 2 as *DPG* increases. When *DPG* = 1.25, the DP-R2L2-MLE performs almost the same as RHCP-R2-MLE. When the relative delay of the MP signal to the LOS signal is within 7 m to 25 m, the DP-R2L2-MLE with *DPG* higher than 1.5 (magenta lines) surpasses the RHCP-R2-MLE (blue line). As *DPG* increases, the improvement becomes larger (the results of DP-R2L2-MLE in Strong LHCP Signal Case 2 are similar to that in Strong LHCP Signal Case 1; hence, the corresponding RMSEs are omitted).

From the results of the two simulations illustrated by Figure 3 and Figure 4, we can conclude that: when there is not enough power in the LHCP channel, i.e., when the power of the MP signal received by the LHCP antenna is lower than −12 dB, dual-polarization antennas hardly help in multipath mitigation based on parameter estimation; when it is higher than −12 dB, dual-polarization antennas contribute to mitigating the short delay multipath.

**Figure 5:**
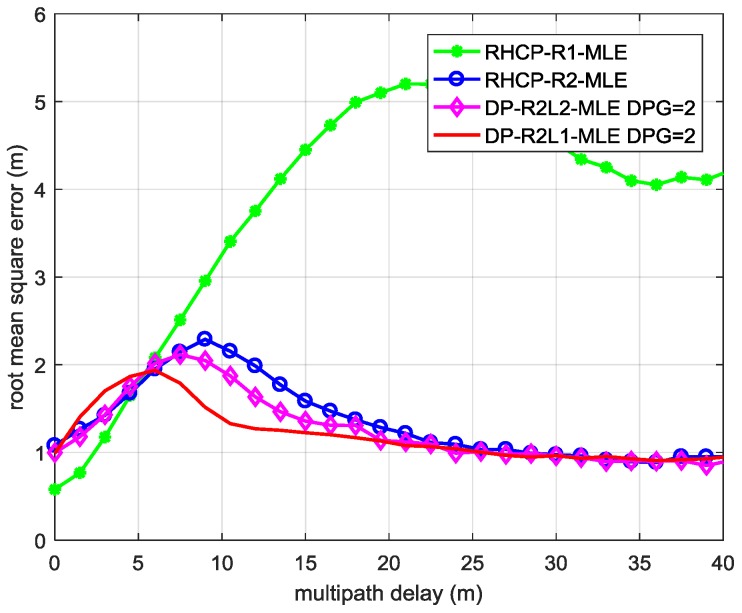
The RMSE of the TOA estimation of LOS signal using MLE with different signal models in the two-path environment defined by Strong LHCP Signal Case 2 when *DPG* = 2.

In Figure 5, we compare the ML estimators in Strong LHCP Signal Case 2 when *DPG* = 2. The DP-R2L1-MLE (red line) outperforms the DP-R2L2-MLE (magenta diamonds line) in the short delay range (except for the range under 5 m). The uncertainty of the TOA of the first MP $\hat{t_{1}}$ in the R2L1 model is greatly reduced compared with that in the R2L2 model, for we can get an almost unbiased estimation of the MP signal from the LHCP channel. The accurate estimation of the MP, in turn, enhances the TOA estimation of the LOS signal because they affect each other mutually [38].

However, when the MP signal is extremely close to the LOS signal (i.e., less than 5 m), DP-R2L1-MLE performs slightly worse than other algorithms. There are two possible reasons. Firstly, the one-path ML estimator actually performs better than the two-path ML estimator for the ill-conditioned parameter estimation problem in the extreme close-in range as concluded in [8,9,24,36]. Secondly, the DP-R2L2-MLE has a high probability of degrading to a one-path ML estimator while DP-R2L1-MLE always works under the two-path model even when the LOS and MP signals are in the extremely close range. To be more specific, the estimated TOAs of MP ($\hat{t_{1}}$) in DP-R2L2-MLE are random within the searching space and the estimated amplitudes of MP ($\hat{A_{1}}$) are often close to zero, whereas the same estimated parameters in DP-R2L1-MLE are neither random nor close to zero, but are close to the true value. This phenomenon indicates that DP-R2L2-MLE often mistakes the noise as the second path and thus, its estimation of the first path is still the LOS and MP composite signal while DP-R2L1-MLE always gives the parameters of two paths that are close to their true values. Consequently, the performance of DP-R2L1-MLE is slightly worse than that of DP-R2L2-MLE when the MP signal is extremely close to the LOS signal. 

From the simulations above, the answer to the fundamental question on the effective conditions of multipath mitigation using a dual-polarization antenna is summarized as follows:

(1) Dual-polarization antenna technology contributes to multipath mitigation when the LHCP antenna receives enough of MP signal power (higher than −12 dB).
(2) If condition (1) is satisfied, dual-polarization antenna technology contributes to mitigating the short delay multipath.

Based on the effective condition, when we design a MLE algorithm for DPMM, we may need a CNR indicator to detect whether the power of the received LHCP signal is high enough to ensure the additional computation load for DPMM is worthwhile. Based on this motivation, we propose an indicator-integrated DP-R2L1-MLE (iiDP-R2L1-MLE) algorithm following the steps below:

(1) Estimate the CNR of RHCP ($CNR_{\mathrm{RHCP}}$) and LHCP ($CNR_{\mathrm{LHCP}}$) channels.
(2) If $CNR_{\mathrm{RHCP}}-CNR_{\mathrm{LHCP}}>threshold$, which means there is not enough power in the LHCP channel, we use the RHCP-R2-MLE to get the TOA estimation of the LOS signal; otherwise we use DP-R2L1-MLE (the threshold we use here is 12 dB based on the effective condition concluded by the simulation results of Figure 3 and Figure 4).

**Figure 6:**
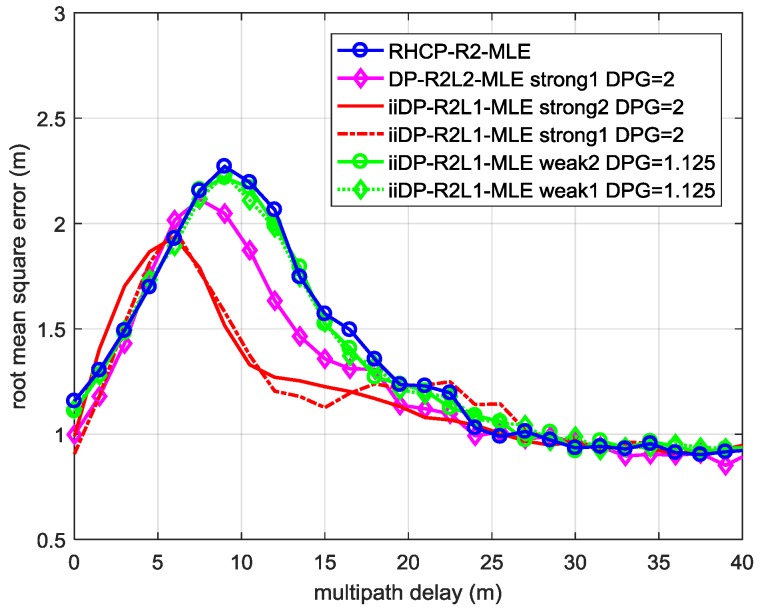
The RMSE of the TOA estimation of the LOS signals of the proposed iiDP-R2L1-MLE in different received signal cases defined in Table 2.

Figure 6 is the performance of the iiDP-R2L1-MLE in different received signal cases defined in Table 2. In Weak LHCP Signal Cases 1 and 2 (*DPG* = 1.25) when there is not enough power in the LHCP channel, the performance of DP-R2L1-MLE (in green diamonds and green circles) degrades to that of RHCP-R2-MLE (in blue circles). In Strong LHCP Signal Case 2 (*DPG* = 2), it performs better than DP-R2L2-MLE and RHCP-R2-MLE within the short delay range from 6 m to 20 m. Comparing the DP-R2L1-MLE in Strong LHCP Signal Case 1 with that in Case 2 where the power of the LOS signal in LHCP channel is different, it deteriorates when the MP signal is about 20 m away from the LOS signal. This result indicates that the increasing of $B_{0}$ does affect the performance of DP-R2L1-MLE for the growing bias of ignoring $B_{0}$ in the R2L1 model. Nevertheless, usually the power of the LOS signal received by the LHCP antenna cannot be too high, and as long as the MP signal dominates in the LHCP channel, ignoring the modeling of the LOS component in the LHCP channel is valid.

To sum up, within the scope of MLE, multipath mitigation can benefit from the dual-polarization antenna technology when the LHCP antenna receives enough power of the MP signal (higher than −12 dB as the power of the received LOS signal in the RHCP channel is normalized to 0 dB) and when short delay MP exists whose relative delay to the LOS signal is less than about 75 ns or 25 m. Under this circumstance, the proposed iiDP-R2L1-MLE algorithm based on the R2L1 model performs better than the conventional RHCP-R2-MLE algorithm.

## 4. Dual-Polarization Multipath Mitigation Algorithm

The two-path ML estimator can be used to assess the theoretical performance of DPMM as analyzed above. However, the heavy computation limits its application in the multiple-path environment. Therefore, in this section, inspired by the iterative MEDLL algorithms [38], we develop a sequential iterative maximum likelihood estimation (SIMLE) algorithm, which has reasonable computational load, to verify our theory. MEDLL is an approximate implementation of the MLE algorithm that uses the locally generated ACFs to match the CCF of the received signal. It is an iterative approximation of Equation (14):

(14) $$\underset{A_{i},\theta_{i},t_{i}}{\min}∥r_{\mathrm{RHCP}}\left(t\right)-\overset{M}{\underset{i=0}{\sum }}A_{i}e^{j\theta_{i}}R_{\mathrm{ss}}\left(t-t_{i}\right)∥$$

where $r_{\mathrm{RHCP}}\left(t\right)=R_{\mathrm{sxI}}\left(t\right)+jR_{\mathrm{sxQ}}\left(t\right)$ is the original CCF of the RHCP channel, $R_{ss}\left(t\right)$ is the ACF of the local code wave, $\left[A_{i},\theta_{i},t_{i}\right]$ are the estimated path parameters and M is the multipath number. *t* is the relative delay to the time of the receiver. The MEDLL algorithm sequentially estimate the path number M based on the estimated SNR or the noise power and decomposes the M-dimensional searching problem to M one-dimensional searching problems to reduce the computational load. Consequently, the mutual relationships between correlated paths are not perfectly considered during iterations. In the one LOS and one reflected path environment, if the relative delay of MP to the LOS signal is in the short delay range, MEDLL often considers the composite signal as the LOS signal and the noise as the MP signal at the beginning of the iteration, which results in a wrong initial TOA estimation for the short delay MP signal. The algorithm with wrong initial status finally converges to a local minimum instead of the global one. Fortunately, when LHCP channel receives enough power of the MP signal, the LHCP channel gives a good initial TOA estimation of $t_{1}$. We can use the CCF of the LHCP channel to assist the iterative MEDLL algorithm in the RHCP channel to improve the short delay multipath mitigation performance, which is the basic concept of our DP-SIMLE algorithm. The proposed DP-SIMLE algorithm minimizes Equation (15):

(15) $$\underset{A_{i},\theta_{i},B_{i},\theta_{i}^{\prime },t_{i}}{\min}\left\{\begin{array}{c} ∥r_{\mathrm{RHCP}}\left(t\right)-\overset{M}{\underset{i=0}{\sum }}A_{i}e^{j\theta_{i}}R_{\mathrm{ss}}\left(t-t_{i}\right)∥+\\ ∥r_{\mathrm{LHCP}}\left(t\right)-\overset{M}{\underset{i=1}{\sum }}B_{i}e^{j\theta_{i}^{\prime }}R_{\mathrm{ss}}\left(t-t_{i}\right)∥ \end{array}\right\}$$

where M is the multipath number. The iterative steps of DP-SIMLE algorithm that simultaneously estimates the path parameters and path number are given below:

(1) Initialization:

Initialize the estimated path number PathNum = 0. Calculate the noise power using the estimated CNR or SNR and the maximum value of the CCF of the RHCP channel, i.e., $SignalPower\;=\underset{t}{\max}\left({\left|r_{\mathrm{RHCP}}\left(t\right)\right|}^{2}\right)$, and $NoisePower\;=\;SignalPower/10^{SNR/10}$. The *t* that maximizes ${\left|r_{\mathrm{RHCP}}\left(t\right)\right|}^{2}$ is denoted as $\hat{t_{\mathrm{RHCP}}}$. Similarly, the *t* that maximizes ${\left|r_{\mathrm{LHCP}}\left(t\right)\right|}^{2}$ is denoted as $\hat{t_{\mathrm{LHCP}}}$. If $\hat{t_{\mathrm{RHCP}}}>\hat{t_{\mathrm{LHCP}}}$, we set $r_{\mathrm{LHCP}}\left(t\right)=0$, and the steps below degenerate to the traditional iterative MEDLL algorithm. This indicator will be triggered in some critical cases which we will talk about in Section 5.5.

(2) Estimate new path:

Obtain the residual CCF by subtracting the generated ACFs of the estimated paths from the original CCF:

$$residual_{\mathrm{RHCP}}\left(t\right)=r_{\mathrm{RHCP}}\left(t\right)-\sum_{i=0}^{PathNum-1}A_{i}e^{j\theta_{i}}R_{\mathrm{ss}}\left(t-t_{i}\right)$$

$$residual_{\mathrm{LHCP}}\left(t\right)=r_{\mathrm{LHCP}}\left(t\right)-\sum_{i=1}^{PathNum-1}B_{i}e^{j\theta_{i}^{\prime }}R_{\mathrm{ss}}\left(t-t_{i}\right)$$

If PathNum<1, $ResidualPower=\underset{t}{\max}\left({\left|residual_{\mathrm{RHCP}}\left(t\right)\right|}^{2}\right)$, otherwise $ResidualPower=\underset{t}{\max}\left({\left|residual_{\mathrm{RHCP}}\left(t\right)\right|}^{2}+{\left|residual_{\mathrm{LHCP}}\left(t\right)\right|}^{2}\right)$. The t that maximizes either equation above is denoted as $t_{\max}$.

If ResidualPower<2×NoisePower, the algorithm stops; otherwise a new path is found and its initial path parameters of the RHCP channel is updated as:

$$t_{PathNum}=t_{\max}$$

$$A_{PathNum}=\left|residual_{\mathrm{RHCP}}\left(t_{\max}\right)\right|$$

$$\theta_{PathNum}=\mathrm{atan}2\left(residual_{\mathrm{RHCP}}\left(t_{\max}\right)\right)$$

If PathNum>0, we update the initial new path parameters in the LHCP channel as:

$$B_{PathNum}=\left|residual_{\mathrm{LHCP}}\left(t_{\max}\right)\right|$$

$$\theta_{PathNum}^{\prime }=\mathrm{atan}2\left(residual_{\mathrm{LHCP}}\left(t_{\max}\right)\right)$$

Increase the estimated path number: PathNum= PathNum+1.

(3) Update the parameters of the estimated paths: (the superscript n=1,2,3,… is the iteration count and subscript j=0,1,…, PathNum−1 is the path index. $\left[A_{i}^{\left(0\right)},\theta_{i}^{\left(0\right)},B_{i}^{\left(0\right)},\theta_{i}^{\prime \left(0\right)},t_{i}^{\left(0\right)}\right]$ are the latest estimated parameters of each path before the iteration in this step).

Obtain the residual CCF by subtracting the generated ACFs of the estimated paths from the original CCF:

$$residual_{j,\mathrm{RHCP}}^{\left(n\right)}\left(t\right)=r_{\mathrm{RHCP}}\left(t\right)-\sum_{i=0,i\neq j}^{PathNum-1}A_{i}^{\left(n-1\right)}e^{j\theta_{i}^{\left(n-1\right)}}R_{\mathrm{ss}}\left(t-t_{i}^{\left(n-1\right)}\right)$$

$$residual_{j,\mathrm{LHCP}}^{\left(n\right)}\left(t\right)=r_{\mathrm{LHCP}}\left(t\right)-\sum_{i=1,i\neq j}^{PathNum-1}B_{i}^{\left(n-1\right)}e^{j\theta_{i}^{\prime \left(n-1\right)}}R_{\mathrm{ss}}\left(t-t_{i}^{\left(n-1\right)}\right)$$

If j<1, it means to update the parameters of the LOS signal:

$$t_{0}^{\left(n\right)}=\underset{t}{\max}\left(\left|residual_{0,\mathrm{RHCP}}^{\left(n\right)}\left(t\right)\right|\right)$$

$$A_{0}^{\left(n\right)}=\left|residual_{0,\mathrm{RHCP}}^{\left(n\right)}\left(t_{0}^{\left(n\right)}\right)\right|$$

$$\theta_{0}^{\left(n\right)}=\mathrm{atan}2\left(residual_{0,\mathrm{RHCP}}^{\left(n\right)}\left(t_{0}^{\left(n\right)}\right)\right)$$

Otherwise update the path parameters of MP signals:

$$t_{j}^{\left(n\right)}=\underset{t}{\max}\left(\left|residual_{j,\mathrm{RHCP}}^{\left(n\right)}\left(t\right)\right|+\left|residual_{j,\mathrm{LHCP}}^{\left(n\right)}\left(t\right)\right|\right)$$

$$A_{j}^{\left(n\right)}=\left|residual_{j,\mathrm{RHCP}}^{\left(n\right)}\left(t_{j}^{\left(n\right)}\right)\right|$$

$$\theta_{j}^{\left(n\right)}=\mathrm{atan}2\left(residual_{0,\mathrm{RHCP}}^{\left(n\right)}\left(t_{j}^{\left(n\right)}\right)\right)$$

$$B_{j}^{\left(n\right)}=\left|residual_{j,\mathrm{LHCP}}^{\left(n\right)}\left(t_{j}^{\left(n\right)}\right)\right|$$

$$\theta_{j}^{\prime (n)}=\mathrm{atan}2\left(residual_{j,\mathrm{LHCP}}^{\left(n\right)}\left(t_{j}^{\left(n\right)}\right)\right)$$

(4) Converge:

If $\frac{\left(A_{0}^{\left(n\right)}-A_{0}^{\left(n-K\right)}\right)}{A_{0}^{\left(n-K\right)}}<0.1\%\;\left(K=3\right),$ or n is larger than the iteration limit, Step (3) converges and then return to Step (2); otherwise n = n+1, and go back to Step (3).

The main difference between the proposed DP-SIMLE and the iterative MEDLL algorithms is the combining of CCFs of both the RHCP and LHCP channels to estimate MP parameters. When it estimates the LOS signal, only the CCF of the RHCP channel is used. The implementation of DP-SIMLE algorithm requires the calculation of path parameters in LHCP channel which results in double the computational load of that of the iterative MEDLL algorithm.

**Figure 7:**
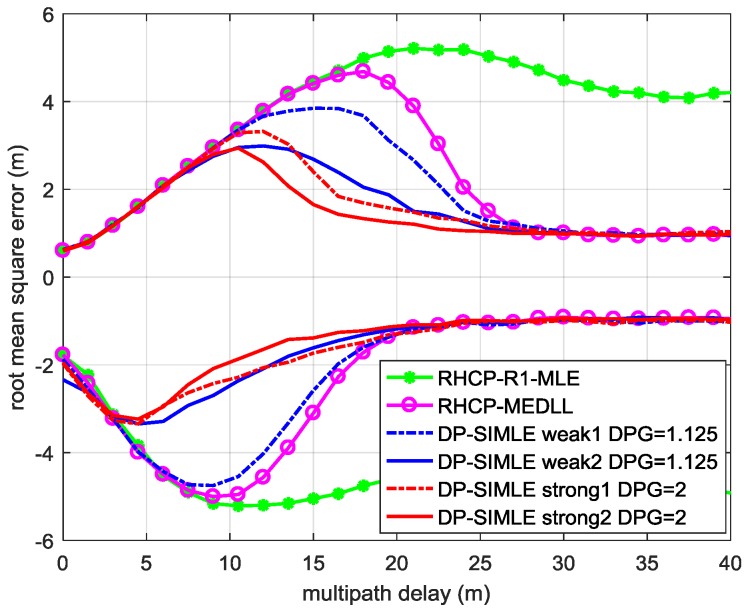
The RMSEs of the TOA estimation of the LOS signal using DP-SIMLE algorithm in different received signal cases defined by Table 2. The lines above zero are the results when constructive multipath is present, and the lines below zeros are that of the destructive multipath. The simulation parameters are the same with that in Section 3. In RHCP channel, the power of the MP signal is 6 dB less than the LOS signal; the CNR is 42 dB-Hz and the integration time is 100 ms.

The RMSEs of $\hat{t_{0}}$ estimated by the DP-SIMLE algorithm in different received signal cases defined by Table 2 are depicted in Figure 7 for theoretical performance evaluation when one constructive (lines above zero) or destructive (lines below zero) multipath is present.

From the results of MC simulations in Figure 7, we can conclude that:

- Comparing the solid line in red with that in magenta, the DP-SIMLE algorithm improves a lot in mitigating multipath whose relative delay to the LOS signal is from 7.5 to 25 m (constructive multipath scenario).
- The increasing RMSE of DP-SMILE algorithm represented by blue lines over that in red indicates that the short delay multipath mitigation performance decreases as the power of the MP received by LHCP antenna decreases.
- The difference between the RMSEs of DP-SIMLE illustrated by solid lines and dashed lines are caused by different powers of the LOS signal in the LHCP channel. Ignoring the received LOS signal by the LHCP antenna does affect the performance of the DP-SIMLE algorithm, and the lower the received power of the MP signal in the LHCP channel is, the stronger the impact.

## 5. Simulation

In this section, we construct a simulation platform to verify the proposed DP-SIMLE algorithm. Under this controlled environment, we simulate the signals received by a dual-polarization antenna such that we could: (1) exclude any other error sources except multipath error; (2) specify the received power of the LOS and MP signals for both RHCP and LHCP antennas; and (3) control the relative delay of the multipath signals to investigate the DP-SIMLE algorithm thoroughly. We firstly use this platform to simulate the one LOS and one reflected path environment to verify the theoretical analysis. Secondly, we investigate how the *DPG* and the power of the LOS signal received by the LHCP antenna affect the performance of DP-SIMLE. Thirdly, we simulate the critical cases when the LHCP channel provides invalid information about the multipath. Finally, we simulate a one LOS with three reflected path environment to test the DP-SIMLE algorithm in the severe multipath environment.

### 5.1. Simulation Platform

**Figure 8:**
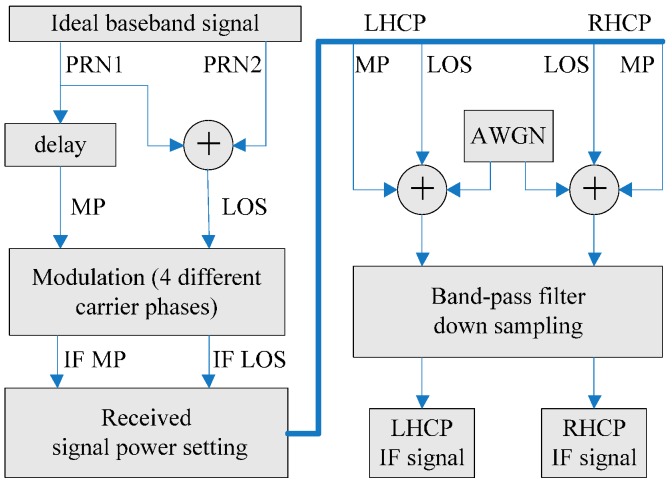
The diagram of the IF signal generator.

**Figure 9:**
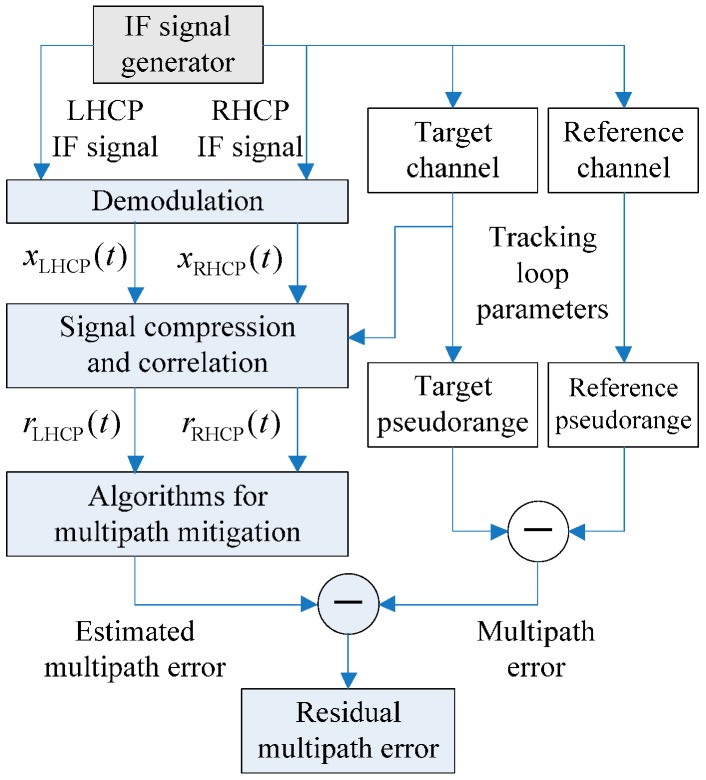
The diagram of the connections between the IF generator, the software receiver and the post-processing unit of simulation platform. The top left part is the IF data generator, the bottom left part is the post-processing unit with multipath mitigation algorithms, and the software receiver is on the right.

The platform consists of three parts, as Figure 8 and Figure 9 illustrate: an intermediate-frequency (IF) signal generator, a software receiver, and a post-processing unit for multipath mitigation. The generator in Figure 8 generates two IF signals to simulate the received signals in RHCP and LHCP channels, respectively. At most four paths can be simulated, with one LOS signal, two static multipath signals and one programmable dynamic multipath signal. The IF signal generator could specify the received signal power and carrier phase for each path in both RHCP and LHCP channels. Pseudorandom Noise (PRN) Code 1 is assigned to the target channel interfered by multipath and PRN Code 2 is assigned to the reference multipath-free channel. The delays and the carrier phases of the LOS signals in both target and reference channels are synchronized. After the rectangular waves of the four signals are generated, the generator modulates them to the IF, superimposes the IF signal of each path to form a composite one, and adds white Gaussian noise to the composite signal. Finally, the oversampled IF signal goes through a band-pass filter and is down-sampled to meet the requirement of the software receiver.

In Figure 9, the software receiver on the right side initiates two channels to track both target (PRN 1) and reference (PRN 2) signals simultaneously. It stores the tracking loop parameters (carrier phase/frequency, code phase/frequency) and the differential pseudorange each time when the tracking loop is updated. The differential pseudorange is obtained by subtracting the pseudorange of the reference channel from that of the target channel. The LOS signals in these two channels are synchronized, thus, the differential pseudorange is the multipath error added with doubled noise which is small enough to be ignored for the narrow bandwidth of the loop filter in the code delay lock loop. The post-processing unit on the left side of Figure 9 uses the tracking loop parameters to apply the signal compression (SC) technology for perfectly matched reception [36] (traditional receiver uses rectangular wave to correlate with the attenuated band-limited baseband signal. Any mismatch of the signal will bring new bias to the multipath error estimation especially when high-resolution algorithm is used). The $r_{\mathrm{RHCP}}\left(t\right)$ or $r_{\mathrm{LHCP}}\left(t\right)$ produced by SC technology has 2001 points within the range between −1 to +1 code chip so that we can ignore the TOA estimation error caused by the searching interval.

The CNR of the signal is 42 dB-Hz, the bandwidth of the band-pass filter is 16 MHz, and the down-sampling rate of the IF I/Q complex value data is 25 MHz. The coherent integration time of the tracking loop in the software receiver is 100 ms, which is also the interval between the consecutive multipath mitigation algorithms. The relative carrier phase of multipath to the LOS signal is set to be a constant value for better comparison of the code multipath mitigation performance. All of the simulations afterwards will use these basic parameters.

### 5.2. One LOS and One Reflected Path Environment

In the simulations below, we compare the performance of the DP-SIMLE algorithm with the RHCP-R1-MLE and the RHCP-MEDLL algorithms, which are designed for the RHCP antenna. In the first simulation, we construct the most basic multipath environment with one reflected signal. Multipath 1 (MP1) shifts 1 m further away from the LOS signal smoothly every 10 s such that we could evaluate the multipath effect within a particular range. We also fix the relative carrier phase of the MP to the LOS signal as 0 or π to obtain the maximum code multipath error to assess the worst case. Table 3 shows the parameters of this two-path scenario. The duration of the simulation is 400 s when MP1 shifts 40 meters away from the LOS signal.

**Figure 10:**
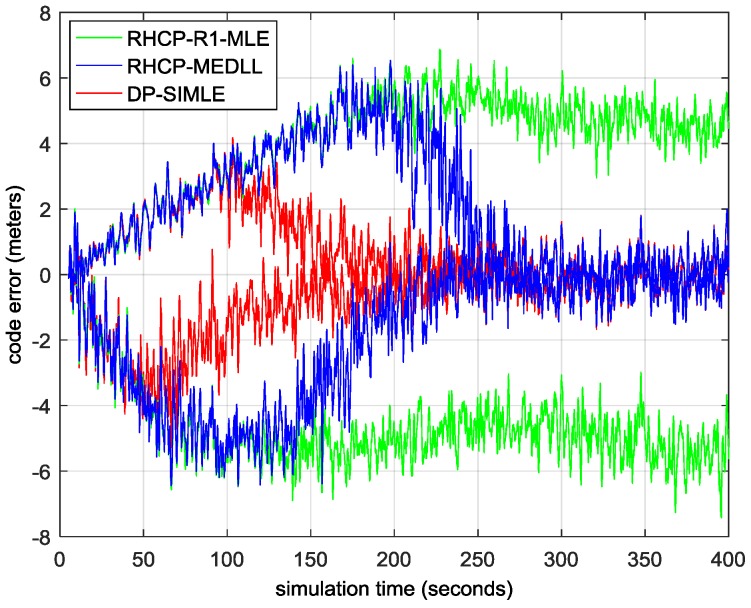
The residual multipath error of the three multipath mitigation algorithms. MP1 shifts 1 m every 10 s, so we can divide the scale of *x* axis by 10 to obtain the true path delay of MP1 relative to the LOS signal conveniently.

**Figure 11:**
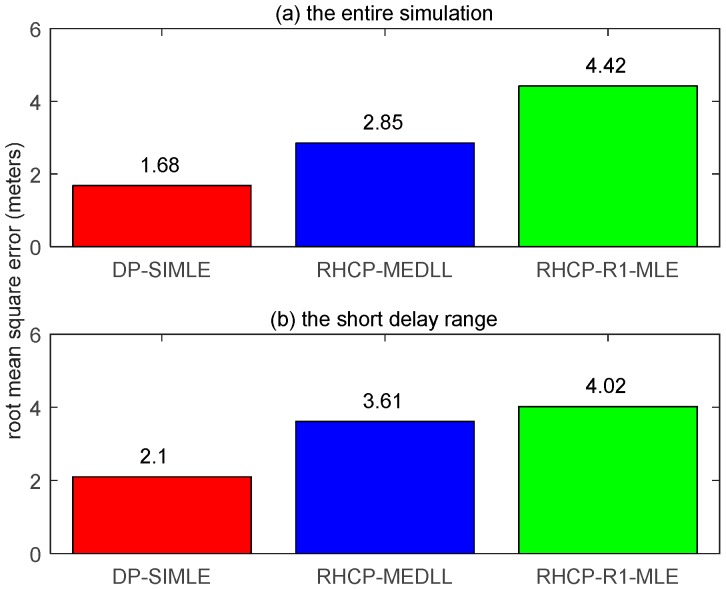
The RMSE of the three multipath mitigation algorithms. (**a**) RMSE in the entire simulation; and (**b**) RMSE in the short delay range.

Figure 10 shows the residual multipath errors of the three algorithms as with the shifting of MP1 when constructive multipath presents (above zero) or destructive multipath presents (below zero). The data of the first 5 s are discarded for the pull-in procedure of the tracking loop. The DP-SIMLE (in red) outperforms the RHCP-MEDLL (in blue) after the MP signal is 10 m (constructive) or 5 m (destructive) further away from the LOS signal. Figure 11 illustrates the RMSE of the proposed DP-SIMLE algorithm: in the entire simulation, the DP-SIMLE algorithm reduces 26% more of the multipath bias than the RHCP-MEDLL algorithm ((2.85 m–1.68 m)/4.42 m); when it comes to the short delay range, the DP-SIMLE algorithm reduces 38% more of the multipath bias than the RHCP-MEDLL algorithm ((3.61 m–2.1 m)/4.02 m).

### 5.3. Dual-Polarization Gain

In this subsection, we decrease the *DPG* from 2 to 1.25 and 1.1. Therefore, the power of the multipath in LHCP channel changes from −6 dB to −12 dB and −16 dB. The resultant RMSEs of this simulation are concluded in Table 4. When $B_{0}^{2}$ is relatively small (−30 dB), the performance of the DP-SIMLE algorithm degrades little as the *DPG* decreases, and even a small amount of the energy of the MP signal (−16 dB) in LHCP channel reduces much of the multipath bias. However, if $B_{0}^{2}$ increases to −15 dB, the degradation becomes more severe as *DPG* decreases. Fortunately, even if *DPG* = 1.1 and $B_{0}^{2}=-15\;\mathrm{dB}$, the DP-SIMLE is still slightly better than RHCP-MEDLL, which is in accordance with the theoretical analysis in Figure 7.

### 5.4. LOS Signal Received by the LHCP Antenna

As we mentioned before, the LOS signal received by the LHCP antenna leads to bias of the estimation of multipath and finally affects the estimated TOA of the LOS signal. Here we increase the power of LOS signal received by the LHCP antenna gradually from −15 dB to −6 dB to evaluate this impact. If the power of LOS signal received by the LHCP antenna reaches −9 dB, it means the AR of the LHCP antenna is 4.7 dB. This level of AR is often measured when the LOS signal comes from a low elevation angle [34]. Table 5 shows the resultant RMSEs of $\hat{t_{0}}$. As we increase the power of the LOS signal in the LHCP channel, the RMSE increases gradually. When the power is −6 dB, which is the same as that of the multipath, the performance of the DP-SIMLE algorithm degrades to that of RHCP-MEDLL. In this critical case, the impact of ignoring the power of the LOS signal in the LHCP channel becomes perceptible. Fortunately, such a high power of the LOS signal in the LHCP channel barely happens if well-designed dual-polarization antennas are used (−6 dB of the LOS power corresponds to the AR of the LHCP antenna to be about 10 dB which is barely seen in a typical circular polarization antenna). To sum up, as long as the multipath dominates the received signal power in the LHCP channel, the DP-SIMLE algorithm using a dual-polarization antenna can outperform the RHCP-MEDLL algorithm using a single-polarization antenna. Therefore, it is verified that ignoring the LOS signal in the LHCP channel is reasonable.

### 5.5. Critical Cases

In real cases, the power of the MP received by a dual-polarization antenna is unpredictable; hence, the LHCP channel may provide invalid information of the MP signal. Table 6 shows two critical cases that may happen when the LHCP channel provides no or false information.

**Figure 12:**
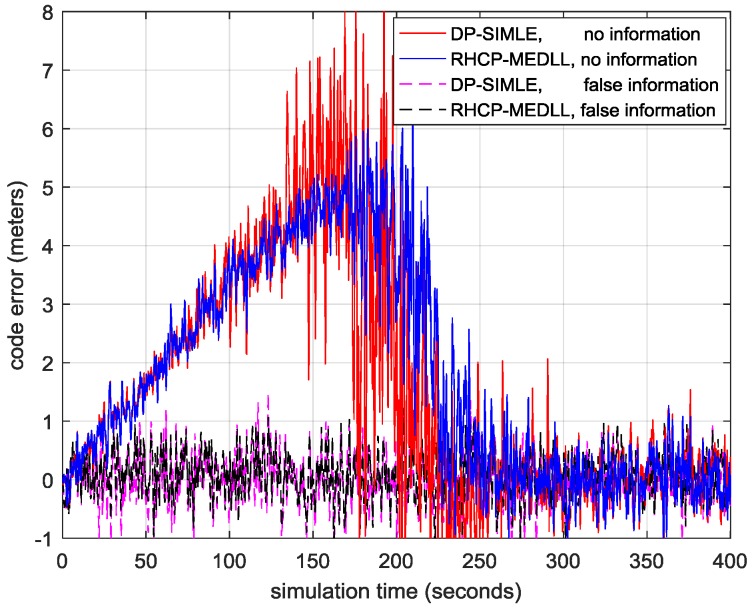
The residual multipath error of DP-SIMLE in some critical cases when the LHCP channel provides invalid information.

In the case described by the left part of Table 6, the LHCP antenna barely receives the MP signal; thus, the merging of the LHCP data provides almost no information of the MP signal, or even disturbance, because of the received LOS signal in the LHCP channel. Figure 12 shows the RMSE of the DP-SIMLE algorithm in this scenario if we disable the indicator in the initialization step. The DP-SIMLE without the indicator has a large variance around 200 s when the relative delay of MP1 to the LOS signal goes to the critical zone where DP-SIMLE is just able to distinguish the LOS signal from the MP signal. Actually, this increased variance is caused by the modeling error in the DP-SIMLE algorithm that ignores the LOS signal in the LHCP channel. However, this phenomenon disappears (1) as the received LOS signal power in LHCP channel $B_{0}^{2}$ decreases (it completely disappears when $B_{0}^{2}$ is less than −30 dB) or (2) when the received MP signal power dominates in the LHCP channel ($B_{1}^{2}\geq B_{0}^{2}$). In order to tackle this problem, we design an indicator to detect whether the MP dominates the LHCP channel by measuring the distance between $\hat{t_{\mathrm{RHCP}}}\;$and$\;\hat{t_{\mathrm{LHCP}}}$ as described in the initialization step in Section 4. Given the CCF of the received LOS and MP compound signal, we regard all of the MP signals as a composite one. In general the $\hat{t}$ that maximizes this CCF is closer to the peak of the ACF of the signal that has higher power. Therefore, $\hat{t_{\mathrm{RHCP}}}$ is close to the TOA of the LOS signal in RHCP channel, and$\;\hat{t_{\mathrm{LHCP}}}$ is close to the composite MP signal if it dominates the LHCP channel. Usually, MP arrives at the antenna later than the LOS signal; hence, if the LHCP antenna receives more of the MP signal power than the LOS signal, $\hat{t_{\mathrm{RHCP}}}<\;\hat{t_{\mathrm{LHCP}}}$; otherwise the received signal may not compatible with the signal model that ignores the LOS signal in the LHCP antenna. Enabling this indicator, the RMSE of the DP-SIMLE in this critical scenario is the same with that of RHCP-MEDLL.

In the case described by the right part of Table 6, there is almost no multipath interference in the RHCP channel; however, the strong MP received by LHCP indicates the existence of MP, which is false information. The result of this critical case in Figure 12 shows that DP-SIMLE performs the same with RHCP-MEDLL in the entire simulation when the LHCP channel provides false information about the multipath.

### 5.6. Four-Path Environment

In severe multipath environments like “urban canyons”, there are possibly more than one multipath. Here we construct a complicated scene with three multipath signals. MP1 is shifting as in the simulations before. MP2 and MP3 are two static multipaths. The power of the LOS signal in the LHCP channel is set to −15 dB. Table 7 shows the parameters of each path.

**Figure 13:**
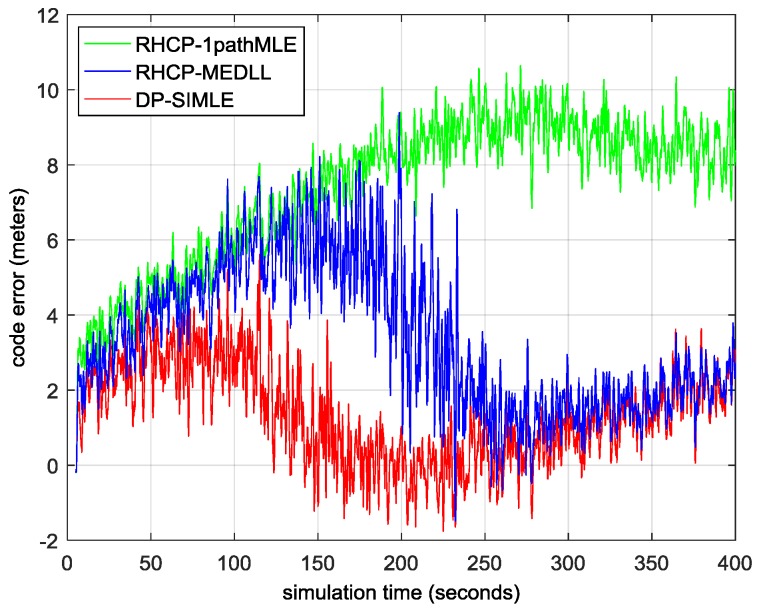
The residual multipath error of different multipath mitigation algorithms in a severe multipath environment with three multipaths.

**Figure 14:**
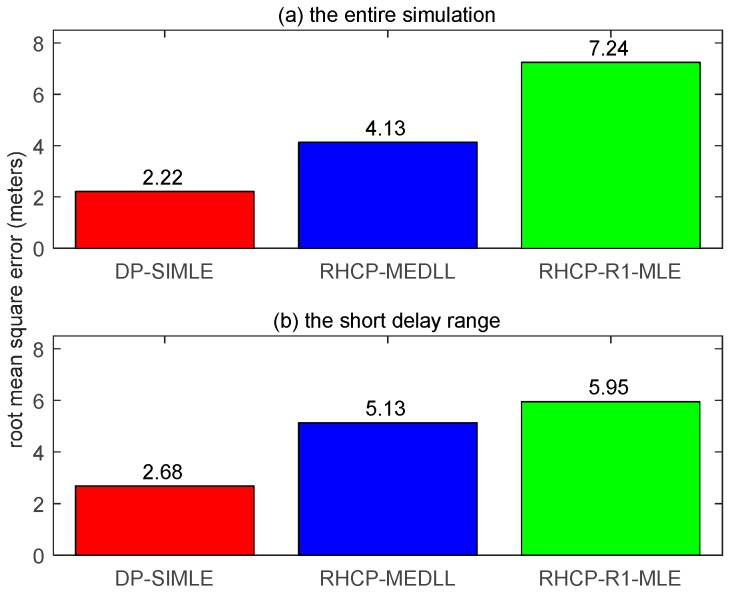
The RMSE of different multipath mitigation algorithms in the four-path simulation. (**a**) The RMSE in the entire simulation; and (**b**) the RMSE in the short delay range.

In this multiple-path environment, though all of the algorithms are biased as Figure 13 shows, the DP-SIMLE algorithm again shows significant improvements in reducing 41% more of the multipath bias in the short delay range ((5.13 m–2.68 m)/5.95 m) as illustrated in Figure 14.

From a series of simulations we can find that: the proposed DP-SIMLE contributes much to mitigating short delay multipath whose relative delay to the LOS signal is less than 25 m when the power of multipath dominates the LHCP channel. Either the LHCP antenna with a bad AR or the low multipath power received by the LHCP antenna degrades the performance of DP-SIMLE. The performance of DP-SIMLE is superior over RHCP-MEDLL in most of the cases. However, DP-SIMLE has its own drawbacks in that its short delay multipath mitigation performance strongly depends on the LHCP antenna’s receiving more of the power of the short delay MP signal than the LOS signal, and it is not easy to ensure this kind of reception every time.

## 6. Conclusions

In this paper we demonstrate the capability of a dual-polarization antenna in multipath mitigation for GNSS receivers. In order to find the answers to the fundamental question about the effective conditions under which the DPMM can outperform the single-polarization one, we firstly model the received signal from a dual-polarization antenna concerning the polarization states of both the antennas and the reflected signals. Based on the typical parameters of the GNSS dual-polarization antenna and the electrical properties of the reflective materials, we analyze the characteristics of the received signals and classify them into different received signal cases. After that we evaluate the theoretical performance of DPMM within the scope of MLE and compare its performance with that of other ML estimators designed for the RHCP antenna in different received signal cases to find that: (1) provided sufficient received power of MP signals for the LHCP antenna (higher than −12 dB relative to the LOS signal in the RHCP channel), the dual-polarization antenna can outperform the single-polarization one in mitigating short delay multipath whose relative delay to the LOS signal is less than 25 m; (2) the greater the power of the MP signal received by the LHCP antenna, the better the performance of DPMM; and (3) when (1) is satisfied, the iiDP-R2L1-MLE algorithm based on the R2L1 model that ignores the LOS signal in LHCP antenna is more appropriate for mitigating the short delay multipath than the DP-R2L2-MLE algorithm based on the R2L2 model. Inspired by the effective conditions and the R2L1 model, we propose the DP-SIMLE algorithm, which takes advantages of the dual-polarization antenna to mitigate short delay multipath. The simulations of the DP-SIMLE algorithm not only verify our theory but also show its superior performance in mitigating short delay multipath over the conventional RHCP-MEDLL algorithm using a RHCP antenna.

## Figures and Tables

**Table 1:** The possible relative power of the received LOS and MP signals by a dual-polarization antenna.

Parameters	Power Level
$A_{0}^{2}$	normalized to 0 dB
$A_{1}^{2}$	−40 dB to 0 dB
$B_{0}^{2}$	−39 dB to −15 dB
$B_{1}^{2}$	−40 dB to −3 dB

**Table 2:** The specific power level of the received LOS and MP signals in the LHCP antenna.

Received Signal Cases	$B_{0}^{2}$	$B_{1}^{2}$ (*DPG* ^1^)
Weak LHCP Signal	–15 dB	−15 dB (1.125), −30 dB (1)
Case 1 (weak1)		
Weak LHCP Signal	–30 dB	−15 dB (1.125)
Case 2 (weak2)		
Strong LHCP Signal	–15 dB	−12 dB (1.25), −9 dB (1.5), −6 dB (2), −3 dB (3)
Case 1 (strong1)		
Strong LHCP Signal	–30 dB	−12 dB (1.25), −9 dB (1.5), −6 dB (2), −3 dB (3)
Case 2 (strong2)		

^1^ The relative power of $A_{1}^{2}$ is −6 dB when calculating *DPG*.

**Table 3:** The parameters of the one LOS and one reflected path signals.

Parameters	LOS	MP1 ^1^
RHCP power	0 dB	−6 dB
LHCP power	−Inf ^2^	−6 dB
path delay	0 m	0 to 40 m ^3^
carrier phase	0	0 or π

^1^ The parameters of MP are relative to the LOS path; ^2^ −Inf means the LHCP antenna does not receive the LOS signal; ^3^ The relative delay of MP1 shifts 1 m further away from the LOS signal smoothly every 10 s.

**Table 4:** The RMSE of the DP-SIMLE algorithm as the DPG decreases.

$B_{0}^{2}$	*DPG* = 2	*DPG* = 1.25	*DPG* = 1.1	RHCP-MEDLL
−30 dB	1.66 m	1.71 m	1.97 m	2.83 m
−15 dB	1.90 m	2.27 m	2.70 m	

**Table 5:** The RMSE of the DP-SIMLE algorithm in the entire simulation as the power of the LOS signal in the LHCP channel increases.

Power of LOS Signal in LHCP Channel					RHCP-MEDLL
−30 dB	−15 dB	−12 dB	−9 dB	−6 dB	
1.66 m	1.91 m	2.09 m	2.36 m	2.71 m	2.83 m

**Table 6:** The parameters of signals when no or false information is provided.

Cases:	No Information		False Information	
Parameters	LOS	MP1	LOS	MP1
RHCP power	0 dB	−6 dB	0 dB	−40 dB
LHCP power	−15 dB	−40 dB	−15 dB	−6 dB
path delay	0 m	0 to 40 m	0	0 to 40 m
carrier phase	0	0	0	0

**Table 7:** The parameters of the signals in the four-path simulation.

Parameters	LOS	MP1	MP2	MP3
RHCP power	0 dB	−6 dB	−9 dB	−15 dB
LHCP power	−15 dB	−9 dB	−13 dB	−12 dB
path delay	0 m	0 to 40 m	15 m	36 m
carrier phase	0	π/4	π/6	−π/6
